# Accuracy of Raman spectroscopy in differentiating brain tumor from normal brain tissue

**DOI:** 10.18632/oncotarget.15975

**Published:** 2017-03-07

**Authors:** Jing Zhang, Yimeng Fan, Min He, Xuelei Ma, Yanlin Song, Ming Liu, Jianguo Xu

**Affiliations:** ^1^ Department of Medical Oncology, State Key Laboratory of Biotherapy, West China Hospital, Sichuan University, and Collaborative Innovation Center for Biotherapy, Chengdu, PR China; ^2^ Department of Neurosurgery, West China Hospital, Sichuan University, Chengdu, PR China; ^3^ West China School of Medicine, West China Hospital, Sichuan University, Chengdu, PR China

**Keywords:** Raman spectroscopy, diagnosis, brain tumors, meta-analysis

## Abstract

Raman spectroscopy could be applied to distinguish tumor from normal tissues. This meta-analysis was conducted to assess the accuracy of Raman spectroscopy in differentiating brain tumor from normal brain tissue. PubMed and Embase were searched to identify suitable studies prior to Jan 1st, 2016. We estimated the pooled sensitivity, specificity, positive and negative likelihood ratios (LR), diagnostic odds ratio (DOR), and constructed summary receiver operating characteristics (SROC) curves to identity the accuracy of Raman spectroscopy in differentiating brain tumor from normal brain tissue. A total of six studies with 1951 spectra were included. For glioma, the pooled sensitivity and specificity of Raman spectroscopy were 0.96 (95% CI 0.94-0.97) and 0.99 (95% CI 0.98-0.99), respectively. The area under the curve (AUC) was 0.9831. For meningioma, the pooled sensitivity and specificity were 0.98 (95% CI 0.94-1.00) and 1.00 (95% CI 0.98-1.00), respectively. The AUC was 0.9955. This meta-analysis suggested that Raman spectroscopy could be an effective and accurate tool for differentiating glioma and meningioma from normal brain tissue, which would help us both avoid removal of normal tissue and minimize the volume of residual tumor.

## INTRODUCTION

The incidence rate and mortality rate of nervous system tumor both rose from 2002 to 2012, according to the latest global cancer statistics [1, 2]. And the incidence rate and mortality rate are higher in more developed areas than in less developed areas, both among males and females [1, 2]. The complete removal of the tumor if possible is the optimal treatment [3]. As to the tumor in the brain, however, the tumor may recur after surgery especially with high World Health Organization (WHO) grade [4, 5]. The predictive factors for recurrence include histological subtype, age, gender and the extent of surgical excision [6, 7]. Though surgical removal of all tumor tissue is important, it is not always possible now. For example, Petrecca et al. found that in patients with glioblastoma failure pattern following complete resection plus radiotherapy and temozolomide was at the resection margin [8]. One of the reasons is that the normal brain tissue must be preserved. Otherwise, it can lead to neurological deficits including impaired motor function, sensory function, memory, vision and audition [9]. Another reason is that the tumor border is always blurred due to the infiltrative growth of tumor tissue. There is no diagnostic technique to define the precise border for tumor excision at present. Therefore, it is urgent to find an effective technique that discriminates brain tumor tissue and normal tissue.

At present, there are conventional diagnostic techniques but all are limited. In situ methods include CT images, MRI, ultrasound sonography and positron emission tomography. They are performed before or after surgery, but the situation may change during the surgery [10]. The incision, dislocation by surgical tools and swelling of the tissue can lead to displacement of the tissue. During surgery, intra-operative MRI requires considerable time and expensive equipment as well. Though florescence-guided surgery and angiography are recommended for malignant tumor, they have inherent difficulties defining the borderline between the low grade and normal tissue [11]. Histopathological diagnosis is purely ex vivo, invasive and time-consuming.

In recent years, many studies were reported on Raman spectroscopy in the diagnosis of various cancers such as tissues of the skin, larynx, breast, esophagus, stomach, cervix and urogenital tract [12–16]. Raman scattering underlies Raman spectroscopic technique. When photons are scattered from a molecule, most photons are elastically scattered, such that the scattered photons have the same energy as the incident photons. A small fraction of the scattered photons have a different frequency from that of the incident photons. Raman spectroscopy tests molecular vibration of asymmetric chemical bonds to detect the inelastic scattering of photons and therefore, provides information on the molecular structure and configuration of the target tissue [17]. Since there are proteomic differences between tumor tissue and normal tissue, Raman spectroscopy can distinguish them at molecular level [17, 18]. Hollon et al. [19] have summarized the Raman spectroscopy techniques in recent years. Also, Raman spectroscopy has several advantages. Water can not disturb the analysis, which remains a problem for other spectroscopic techniques. Also, the fiber-optic probe allows spatial flexibility to achieve non-destructive and non-invasive collection of spectrum [20]. Furthermore, the spectra can be rapidly processed and a result can be offered in real-time during the surgery.

The first studies using Raman spectroscopy for neuro-oncologic applications were from Mizuno et al [21]. At that time, the less-advanced instrumentation and processing software didn't allow it to be a surgical tool. Recently, many studies have been published to examine the accuracy of Raman spectroscopy in distinguishing brain tumor from normal tissues and to map the spectra of different sections of brain tissue [22, 23]. However, these studies were inconclusive because of insufficient sample and different diagnostic algorithms. The aim of this meta-analysis was to systematically evaluate the accuracy of Raman spectroscopy for discriminating brain tumor and normal brain tissues.

## RESULTS

### Study identification

**Figure 1:**
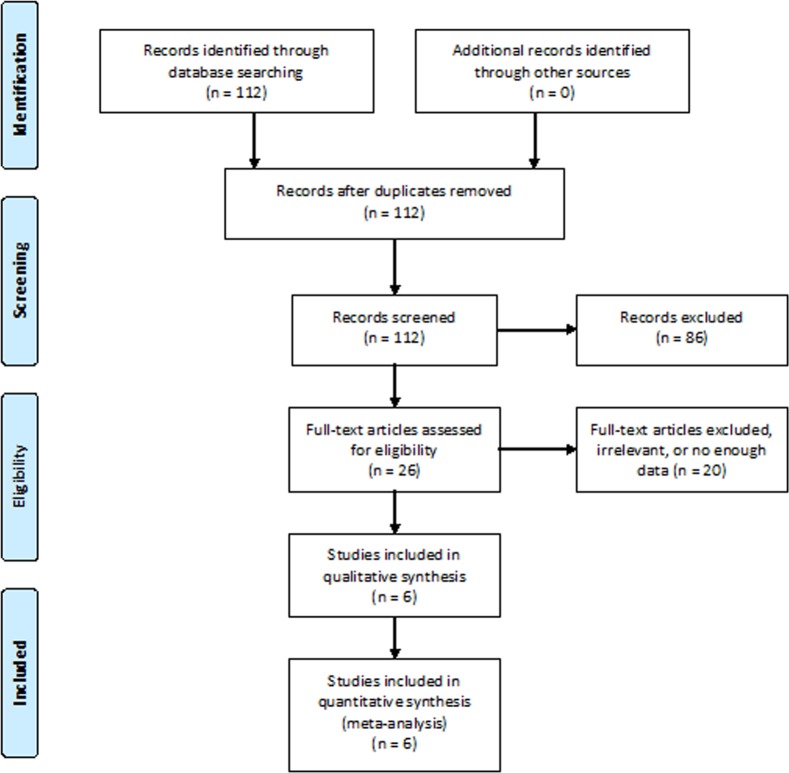
Literature search and selection

The initial literature research yielded 112 articles. According to the selection criteria, 26 relevant articles were selected and reviewed in full-text for more detailed information. 12 articles were irrelevant and 8 had insufficient details to reconstruct the 2×2 table. Ultimately, 6 studies [17, 18, 24–27] were retrieved according the inclusion criteria. The study selection process was shown in Figure 1.

### Description of included studies

The detailed characteristics of the 6 studies were shown in Table 1. All the articles were published between 2005 and 2015, with 5 of them during the last five years. These studies were operated in 5 different countries. The number of the included patients varied from 7 to 28. The number of tissues involved in each study varied from 3 to 60. The number of the spectra retrieved varies from 16 to 1198. The total number of spectra was 1951, with an average of 325. In these 6 eligible studies, 3 studies identified glioma and 2 studies identified meningioma. The rest study identified both glioma and meningioma. The sample types included ex vivo (during the neurosurgical procedure or after that) and *in vivo* (only in one study). Three out of 6 articles involved cross-validation. Several diagnostic algorithms were utilized to discriminate spectra of different brain tissues. Raman spectra of the tissue were obtained by 2 types of Raman spectroscopy in these studies.

**Table 1:** Baseline characteristics of included studies

First Author	Year	Country	N1	N2	N3	N4	Tumor type	Mean age	Sample type	Cross validation	Diagnostic algorithm	Raman spectroscopy
Koljenovic	2005	Netherlands	20	20	38	115	meningioma	59	ex vivo	Yes	LDA	NIRS
Leslie	2012	USA	28	24	60	296	glioma	—	ex vivo	Yes	SVMA	CRM
Zhou	2012	China	7	3	3	16	meningioma	27-56 (range)	ex vivo	No	PCA & SVMA	CRM
Aguiar	2013	Brazil	—	—	6	165	glioma & meningioma	—	ex vivo	No	PCA	NIRS
Kalkanis	2014	USA	17	17	40	1198	GBM	63.9 (GBM), 31.8 (normal)	ex vivo	No	DFA	CRM
Jermyn	2015	Canada	17	15	—	161	glioma	53	*in vivo*	Yes	BTC	NIRS

N1 number of total patients, N2 number of patients in test group (not training), N3 number of tissues, N4 number of spectra, GBM glioblastoma multiforme, LDA linear discriminant analysis, SVMA support vector machine analysis, PCA principal component analysis, DFA discriminant function analysis, BTC boosted trees classification, NIRS near infrared Raman spectrometer, CRM confocal Raman microscope.

### Diagnostic accuracy

#### Glioma group

**Figure 2:**
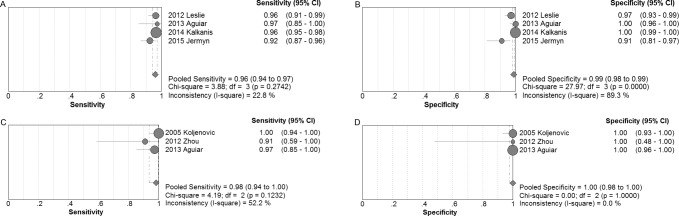
Individual study and pooled estimates of sensitivity and specificity and their 95% CIs of Raman spectroscopy to differentiate glioma (**A** and **B**) and meningioma (**C** and **D**) from normal tissues.

**Figure 3:**
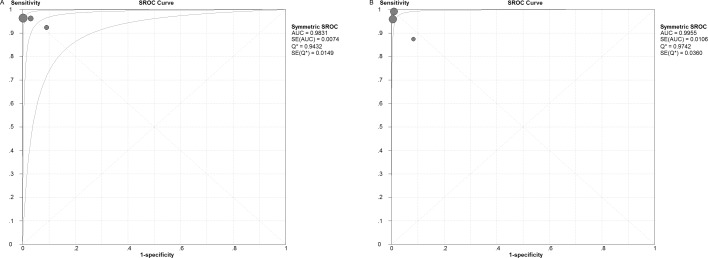
Summary receiver operating characteristics (SROC) curve of Raman spectroscopy to differentiate glioma (**A**) and meningioma (**B**) from normal tissues.

Four studies [17, 18, 24, 26] examined glioma. The pooled sensitivity and specificity of Raman spectroscopy for discriminating glioma and normal brain tissues were 0.96 (95% CI 0.94-0.97) and 0.99 (95% CI 0.98-0.99), respectively. The forest plots were shown in Figure 2. The pooled PLR and NLR were 62.09 (95% CI 8.66-445.29) and 0.05 (95% CI 0.03-0.08), respectively. The DOR was 1345.65 (95% CI 136.55-13260.52), demonstrating high accuracy. The SROC curve analysis was used to summarize overall diagnostic accuracy. The AUC was 0.9831. The SROC curve was shown in Figure 3.

#### Meningioma group

Three studies [18, 25, 27] examined meningioma. The pooled sensitivity and specificity of Raman spectroscopy for discriminating meningioma and normal brain tissues were 0.98 (95% CI 0.94-1.00) and 1.00 (95% CI 0.98-1.00), respectively. The forest plots were also shown in Figure 2. The pooled PLR and NLR were 56.34 (95% CI 9.46-335.35) and 0.05 (95% CI 0.01-0.24), respectively. The DOR was 1527.83 (95% CI 70.47-33123.41), also demonstrating very high accuracy. The SROC curve was also performed to summarize overall diagnostic accuracy. The AUC was 0.9955. The SROC curve was shown in Figure 3.

### Assessment of study quality

Two reviewers evaluated methodological quality for each study according to the QUADAS guidelines independently. All QUADAS items were used to evaluate the eligible articles. Table 2 shows the results of the evaluation of each study.

**Table 2:** Quality assessment of included studies using QUADAS questionnaire

Author	Q1	Q2	Q3	Q4	Q5	Q6	Q7	Q8	Q9	Q10	Q11	Q12	Q13	Q14	Score
2005 Koljenovic	Y	N	Y	Y	Y	Y	Y	Y	Y	Y	Y	Y	N	U	11
2012 Leslie	Y	Y	Y	Y	Y	Y	Y	Y	Y	Y	Y	Y	N	U	12
2012 Zhou	Y	N	Y	Y	Y	Y	Y	Y	Y	Y	Y	Y	N	U	11
2013 Aguiar	Y	N	Y	Y	Y	Y	Y	Y	Y	Y	Y	Y	N	U	11
2014 Kalkanis	Y	N	Y	Y	Y	Y	Y	Y	Y	Y	Y	Y	N	U	11
2015 Jermyn	Y	Y	Y	Y	Y	Y	Y	Y	Y	Y	Y	Y	N	U	12

QUADAS Quality assessment of diagnostic accuracy studied, Y yes, N no, U unclear.

Q1. Was the spectrum of patients representative of the patients who will receive the test in practice? Q2. Were selection criteria clearly described? Q3. Is the reference standard likely to correctly classify the target condition? Q4. Is the time period between reference standard and index test short enough to be reasonable? Q5. Did the whole sample, or a random selection of the sample, receive verification using a reference standard of diagnosis? Q6. Did patients receive the same reference standard regardless of the index test result? Q7. Was the reference standard independent of the index test (i.e. the index test did not form part of the reference standard)? Q8. Was the execution of the index test described in sufficient detail to permit replication of the test? Q9. Was the execution of the reference standard described in sufficient detail to permit its replication? Q10.Were the index test results interpreted without knowledge of the results of the reference test? Q11. Were the reference standard results interpreted without knowledge of the results of the index test? Q12. Were the same clinical data available when test results were interpreted as would be available when the test is used in practice? Q13. Were interpretable/intermediate test results reported? Q14. Were withdrawals from the study explained?

### Publication bias

**Figure 4:**
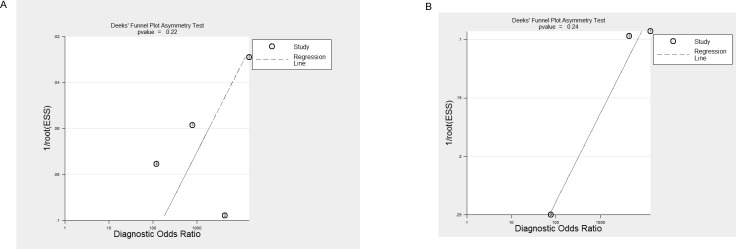
Deeks' funnel plots indicating no publication bias for glioma (**A**, *p* = 0.22) and meningioma (**B**, *p* = 0.24) groups.

The Deeks' funnel plot asymmetry tests demonstrated that no significant publication bias was found in both glioma group (*p* = 0.22) and meningioma group (*p* = 0.24). The funnel plots were shown in Figure 4.

## DISCUSSION

This meta-analysis was conducted to assess the accuracy of Raman spectroscopy in differentiating brain tumor from normal brain tissue. This research for the first time summarized the evidence on the accuracy of Raman spectroscopy in the detection of glioma and meningioma. For glioma, the pooled sensitivity and specificity of Raman spectroscopy were 0.96 and 0.99, respectively. The AUC was 0.9831. For meningioma, the pooled sensitivity and specificity were 0.98 and 1.00, respectively. The AUC was 0.9955. Based on the principle mentioned in the methods section, we can conclude that Raman spectroscopy is a viable candidate for differentiating glioma and meningioma from normal brain tissue.

Raman spectroscopy is a promising tool as intraoperative neurosurgical guidance. First, it requires no special staining or any preparation, which makes it possible for the diagnosis in real-time and to be able to minimize the disruption to neurosurgical workflow [24]. Second, it only takes a few minutes to obtain an accurate diagnostic result through Raman while the analysis of tissue used to require hours or even days through traditional analytic technique [28]. Third, Raman spectroscopy causes no harm to the patients, which differs from the traditional biopsy [29]. Besides, the high accuracy of Raman decreases the number of expensive tests, such as immunohistochemical staining, fluorescent in situ hybridization, electron microscopy or karyotyping, which are prescribed to guarantee the correct diagnosis [26]. Furthermore, the handheld Raman probe is small and easy to use during the surgery [30]. Raman spectroscopy can both quickly and effectively detect and analyze brain tissue *in vivo* as well as providing accurate tumor margin assessment by rapidly scanning [26]. With clear knowledge of margin, it contributes to avoid removal of normal tissue and to minimize the volume of residual tumor which poses a considerable impact on patient survival [24].

Besides the intraoperative use, Raman-guided biopsy with high accuracy contributes to reduce the incidence of a second stereotactic biopsy procedure when no representative tumor tissue is found for the first time [31]. Though second stereotactic biopsy rarely happened, stereotactic biopsy can cause hemorrhage and direct trauma [29]. Raman technique can also be applied to identification of location during radiation therapy [32].

Compared with neurosurgical microscopy, Raman spectroscopy does have a restricted field of view. It may be solved by involving complementary imaging technique. Besides, Raman spectroscopy requires proper illumination setup to limit extraneous light sources in measured signal. When we design the operating room for low-intensity spectroscopic signal, engineering solution may be needed [24].

This study also had several limitations. First, this meta-analysis is based on a limited number of studies. Though the number of spectra involved in is large (1951 spectra), more studies are needed. Second, the patient size in each study was small and the numbers of spectra differed sharply among the included studies and this variability might have affected the outcome. Thirdly, the majority of the studies used ex vivo tissue. To prove whether Raman spectroscopy is an optimal diagnostic tool or not, more studies involving *in vivo* technique are needed. Furthermore, different techniques of Raman spectroscopy, and multiple algorithms were used in the included studies. Finally, the publication bias was a major concern for all meta-analysis. In our meta-analysis, though no publication bias was found (p>0.05), it should be noted that any meta-analysis could not completely exclude biases. Therefore, more studies with more patients examined *in vivo* are needed.

In conclusion, our study suggested that Raman spectroscopy could be an effective and accurate tool for differentiating glioma and meningioma from normal brain tissue. The application of this promising novel method would improve the accuracy of brain tumor surgery in the future, by both avoiding removal of normal tissue and minimizing the volume of residual tumor. However, more studies are warranted to verify that and more efforts are still needed to improve this equipment and better serve clinical work.

## MATERIALS AND METHODS

### Literature search

PubMed and Embase were searched to identify suitable studies on Jan 1st, 2016, and no start date limit was applied. The search terms were ‘brain neoplasms’, ‘spectrum analysis, Raman’, and ‘diagnosis’. Reference lists of relevant articles were also searched. No language restriction was applied.

### Study selection criteria

Two reviewers independently determined study eligibility. Disagreements were adjudicated by a third reader.

The studies were selected on the basis of the following criteria: 1) only human tissue used in the experiments; 2) Raman spectroscopy was used as a diagnosis tool to distinguish tumor and normal tissues; 3) used histopathology as golden standard; 4) provided with detailed data to construct a 2×2 contingency table for true positive (TP), false positive (FP), true negative (TN) and false negative (FN). If the four values were not reported, we calculated those using indexes such as sensitivity and specificity. Corresponding author were contacted for the detailed data if no enough data was available.

Excluded criteria: 1) used animal tissues; 2) histopathology was not the reference standard; 3) included less than 10 spectra samples; 4) without sufficient calculable data; 5) duplicated reports, conference abstracts or studies based on the same study.

### Data extraction

Two investigators extracted the data independently and disagreements were resolved by consensus. First author, year of publication, country, the number of patients, the age of patients, the number of samples, tumor type, methodological and technical data, numbers of TP, FP, TN, and FN were extracted from each study.

### Quality assessment

The quality of each study was assessed by using the Quality Assessment of Diagnostic Accuracy Studies (QUADAS) guidelines, which is an established, evidence-based tool for systematic reviews of diagnostic studies designed for diagnostic accuracy [33].

### Statistical methods

Using the extracted data of TP, TN, FP, and FN, the pooled sensitivity, specificity, positive and negative likelihood ratios (LR), and diagnostic odds ratio (DOR), with 95% confidence intervals (CI), were calculated based on bivariate generalized linear mixed modeling [34]. Meta-Disc version 1.4 statistical software was used.

Furthermore, summary receiver operator characteristics (SROC) curves were constructed to examine the relationship between sensitivity and specificity. And the area under the curve (AUC) was calculated to assess the overall performance of Raman spectroscopy. In general, a diagnostic tool is regarded excellent when AUC values were between 0.9-1, good when AUC values were between 0.8-0.9, fair when AUC values were between 0.7-0.8, poor when AUC values were between 0.6-0.7 and failed when AUC values were between 0.5-0.6 [35]. The SROC curves were also performed by Meta-Disc version 1.4.

### Publication bias

Publication bias was assessed using Deeks' funnel plot asymmetry test (p<0.05 was considered that potential publication bias exits). The Deeks' funnel plot asymmetry test was performed by Stata 11.0.
